# Diversity and structure of feather mite communities on seabirds from the north–east Atlantic and Mediterranean Sea

**DOI:** 10.1038/s41598-023-30858-8

**Published:** 2023-03-23

**Authors:** Laura M. Stefan, Wolf Isbert, Elena Gómez-Díaz, Sergey V. Mironov, Jorge Doña, Karen D. McCoy, Jacob González-Solís

**Affiliations:** 1 Institut de Recerca de La Biodiversitat (IRBio), Departament de Biologia Evolutiva, Ecologia i Ciències Ambientals, Universitat de Barcelona, Av. Diagonal 643, 08028 Barcelona, Spain; 2 Department of Cellular and Molecular Biology, National Institute of Research and Development for Biological Sciences, Splaiul Independentei 296, 060031 Bucharest, Romania; 3 Université de Montpellier- CNRS-IRD, UMR MIVEGEC, 900 Rue Jean-François Breton, 34090 Montpellier, France; 4 Unidad de Zoología Marina, Institut Cavanilles de Biodiversitat i Biologia Evolutiva, Parc Científic, Universitat de València, PO Box 22085, 46071 Valencia, Spain; 5 Alfred Wegener Institute Helmholtz Centre for Polar and Marine Research, 27570 Bremerhaven, Germany; 6 Instituto de Parasitología y Biomedicina López-Neyra, Consejo Superior de Investigaciones Científicas (CSIC), Av. del Conocimiento 17, 18016 Granada, Spain; 7 Zoological Institute, Russian Academy of Sciences, Universitetskaya Embankment 1,, 199034 Saint Petersburg, Russia; 8 Illinois Natural History Survey, Prairie Research Institute, University of Illinois at Urbana-Champaign, 1816 S. Oak St., 61820 Champaign, IL USA; 9 Departamento de Biología Animal, Univerdidad de Granada, Avenida de Fuente Nueva S/N, E-18071 Granada, Spain

**Keywords:** Ecology, Zoology

## Abstract

The richness and structure of symbiont assemblages are shaped by many factors acting at different spatial and temporal scales. Among them, host phylogeny and geographic distance play essential roles. To explore drivers of richness and structure of symbiont assemblages, feather mites and seabirds are an attractive model due to their peculiar traits. Feather mites are permanent ectosymbionts and considered highly host-specific with limited dispersal abilities. Seabirds harbour species-rich feather mite communities and their colonial breeding provides opportunities for symbionts to exploit several host species. To unravel the richness and test the influence of host phylogeny and geographic distance on mite communities, we collected feather mites from 11 seabird species breeding across the Atlantic Ocean and Mediterranean Sea. Using morphological criteria, we identified 33 mite species, of which 17 were new or recently described species. Based on community similarity analyses, mite communities were clearly structured by host genera, while the effect of geography within host genera or species was weak and sometimes negligible. We found a weak but significant effect of geographic distance on similarity patterns in mite communities for Cory’s shearwaters *Calonectris borealis*. Feather mite specificity mainly occurred at the host-genus rather than at host-species level, suggesting that previously inferred host species-specificity may have resulted from poorly sampling closely related host species. Overall, our results show that host phylogeny plays a greater role than geography in determining the composition and structure of mite assemblages and pinpoints the importance of sampling mites from closely-related host species before describing mite specificity patterns.

## Introduction

The composition and structure of symbiont assemblages (parasites, commensals or mutualists) can be shaped by a wide array of variables including symbiont intrinsic factors (i.e., host specificity)^1,2^, extrinsic host features (i.e., phylogeny, morphological characteristics, behaviour)^3–5^ or ecological factors (i.e., host diet, host habitat, geographic distance between sampling localities)^6–9^. For example, we would expect phylogenetically related hosts to harbour similar symbiont assemblages that were acquired through co-speciation or host-switching. In the latter case, similarity would be explained by the fact that related hosts have more characteristics in common than unrelated species, such as similar living conditions for symbionts or similar defense systems^5^. This similarity has been found in symbionts of freshwater fishes, rodents and primates^5,9,10^. However, in other studies host phylogeny failed to fully explain similarity in symbiont communities among hosts, indicating that other extrinsic factors, such as environmental conditions, host ecology and geographic distance could also play a role^11,12^. Among studies evaluating similarity of symbiont richness to date, most have focused on endoparasites, with less attention given to ectoparasites/ectosymbionts.

Geography may also play an important role in defining symbiont community richness, with an expectation of decreasing similarity with increasing distance. The predicted decay in similarity has been reported for various parasite groups from a wide range of host taxa including fishes^6,8^, birds^13,14^, mammals^15,16^ and invertebrates^17^. Several mechanisms have been proposed to explain this pattern, such as environmental structure (i.e., decreasing similarity in environmental features with increasing distance), spatial configuration of the landscape (i.e., major geographic barriers limiting dispersal and leading to reduced community similarity) and limited dispersal potential of the species^18^.

Feather mites (Astigmata: Pterolichoidea and Analgoidea) are the most common and diverse group of permanent ectosymbionts associated with birds^19,20^. Most species live on the external surface of feathers, with specific adaptations to feather morphology^20,21^. Almost all avian orders have their own specific feather mite fauna, inhabiting one or a very few closely-related host species^22,23^. However, some mite species live on different host genera and even on different host families or orders^20,23^. In addition, only a small part of feather mite diversity has been described so far^22^. Thus, it is important to characterize the entire mite community of interest before drawing conclusions on host-specificity.

Co-speciation has been considered to be the main process driving feather mite diversification and community structuring, under the assumption that feather mites are highly specialized, host-specific symbionts^23,24^. Surprisingly, molecular studies have shown that host-switching, rather than co-speciation, may be the main process defining evolutionary patterns of divergence, even for these symbionts with limited dispersal potential^23,24^. Many studies on feather mite-bird systems have examined the prevalence/abundance patterns of these ectosymbionts on passerine hosts in relation to different factors. Intraspecific and interspecific studies have shown that feather mite abundance correlates with various host traits (i.e., body size, body mass, body condition, size of the uropygial gland, plumage coloration, sociality;^25–27^) and/or with environmental variables (i.e., salt concentration in the air;^28^). Mite prevalence has also been shown to positively correlate with winter sociality (i.e., birds living in flocks during the non-breeding season;^29^), but was unrelated to bird size, body mass or migratory behaviour. Taken together, these results suggest that feather mite abundance may be mostly shaped by habitat-associated conditions (i.e., host body environment, air temperature, season, humidity), whereas prevalence is more linked to mite transmission opportunities (i.e., winter sociality, phoresis)^30,31^. Given the diversity of results from molecular and ecological data to date, the main factors driving overall community structure of feather mites remain difficult to discern.

Seabirds, particularly Procellariiformes, harbour diverse ectosymbiont communities. They are known to be exploited by hard and soft ticks, blood-feeding mites, feather mites, fleas, lice and hippoboscid flies^20,32^. In addition, seabirds are highly mobile pelagic species, with a global distribution, and most of them breed sympatrically in large, mixed species colonies on isolated islands, features that should promote ectosymbiont dispersal within and among host species. However, they also show strong interannual fidelity and natal philopatry to their breeding sites, which means that most contact transmission may take place among related individuals^33,34^. Feather mite species richness seems to be particularly high on these birds^35–39^, with *Microspalax*, *Zachvatkinia* and *Brephosceles* being the most common and diverse genera^35,40,41^.

The Mediterranean Sea and Northeast Atlantic Ocean are key breeding areas for a number of procellariiform species of the genera *Calonectris*, *Puffinus*, *Bulweria*, *Pterodroma*, *Pelagodroma* and *Hydrobates*, including many closely-related and regionally endemic species, all of them harboring rich feather mite communities^42^. Indeed, six new mite species were described based on the feather mite surveys used for the present study^43–45^. In addition, feather mites inhabiting sympatric seabird species in the Cape Verde archipelago were found to exhibit strong host-associated structure^42^. These different attributes render this group of birds ideal for studying the factors structuring ectosymbiont communities.

In this context, the aim of the present study was to examine the influence of host phylogeny and geography on the composition and structure of feather mite communities found on the procellariform seabirds of the Mediterranean Sea and the archipelagos of the north-east Atlantic Ocean. Based on our current knowledge of feather mites and their avian hosts, we expected to find an increase in similarity with host relatedness given that feather mites are permanent ectosymbionts and are considered to transmit primarily from parents to offspring. Second, we expected to find a decrease in mite community similarity with increasing geographic distance between breeding colonies based on the fact that nearby colonies are more ecologically similar and/or because feather mite dispersal is more likely among neighbouring birds.

## Materials and methods

### Seabird sampling

**Figure 1:**
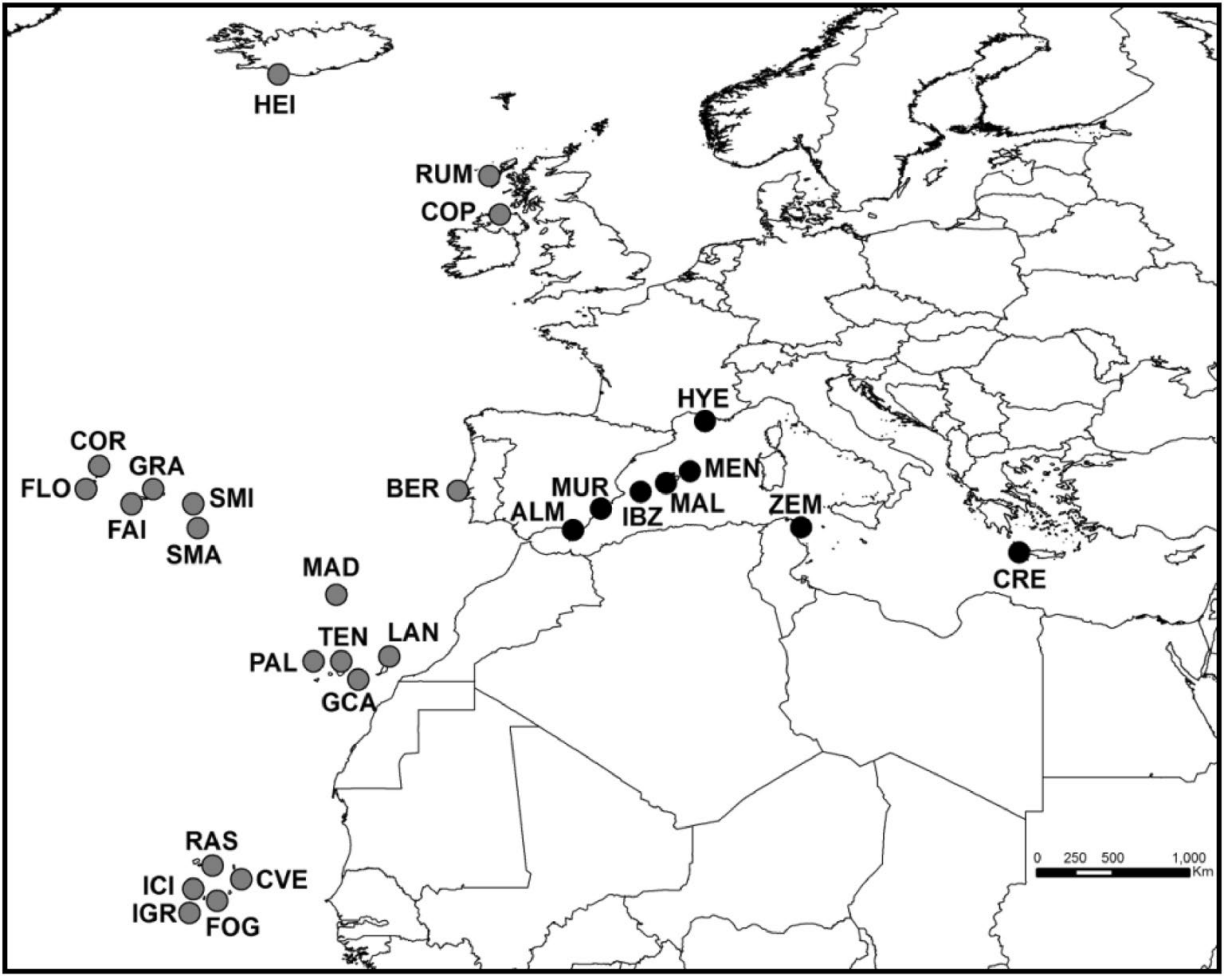
Map of the study area showing location of the 28 sample sites for feather mites on procellariiform seabird species across the north-east Atlantic Ocean (grey circles) and Mediterranean Sea (black circles). Abbreviations of the sample sites are shown in Supplementary Table S1.

In total, we collected and examined feather mites from 964 sampled seabirds, covering 11 host species and 28 geographic locations. Host birds included 11 procellariiform species, nine belonging to Procellariidae (Scopoli´s shearwater, *Calonectris diomedea*; Cory’s shearwater, *Calonectris borealis*; Cape Verde shearwater, *Calonectris edwardsii*; Manx shearwater, *Puffinus puffinus*; Mediterranean shearwater, *Puffinus yelkouan*; Macaronesian shearwater, *Puffinus baroli*; Boyd’s shearwater, *Puffinus boydi*; Bulwer’s petrel, *Bulweria bulwerii* and Cape Verde petrel, *Pterodroma feae*), and two belonging to Hydrobatidae (Band-rumped storm-petrel, *Hydrobates castro* and European storm-petrel, *Hydrobates pelagicus*). The 28 sampled sites included eight breeding colonies across the Mediterranean Sea and 20 across the north-east Atlantic Ocean (Fig. 1, Supplementary Fig. S1 online). Certain colonies were found in an area, defined here as a group of colonies within an archipelago (e.g., Balearic Islands, Azores Islands, Canary Islands and Cape Verde Islands). Colonies were also defined at a regional scale, a region being a larger geographic zone that includes several colonies and areas (e.g., Western Mediterranean—WM, Eastern Mediterranean—EM, Northern NE Atlantic—NNEA, Central NE Atlantic—CNEA and Southern NE Atlantic—SNEA). Bird capture and mite sampling were performed in accordance with good animal practices, as defined by the current European legislation and under permission from corresponding governmental authorities (Cabildo Insular de Gran Canaria y Lanzarote, Gobierno de Canarias, Secretaria Regional do Ambiente da Região Autónoma dos Açores, Parque Nacional do Madeira, Direcção Geral do Ambiente from Cape Verde, Govern de les Illes Balears, Junta de Andalucia, Región de Murcia—Consejeria de Agricultura y Agua). It also followed the recommendations of the ARRIVE guidelines (https://arriveguidelines.org). Birds were conscious during sampling and immediately released after handling with no apparent detrimental effects. Birds were not anesthetized and/or unconscious on any occasion. From 2003 to 2012, we collected feather mites from adult birds using the dust-ruffling method^46^ for all species, except for Cory´s and Scopoli’s shearwaters due to their large body size. For these two host species, we collected mites by visual inspection of primary and body feather barbs. Nearly all individuals of these two species harboured at least one mite species. However, as visual detection is more restrictive than dust-ruffling, the comparison of species prevalence between the two methods should be treated with caution. All samples were stored in absolute ethanol at − 20 °C for subsequent morphological identifications.

### Feather mite identification and deposition of mite specimens

Mites were cleared in lactic acid for 24 h, mounted on microscope slides in PVA medium and examined using a Leica DM 5000B light microscope. Mites were identified using specific keys for avenzoariids^35,36^, xolalgids^47^ and alloptids^40,41,48^. Feather mite specimens were deposited in the Zoological Institute of the Russian Academy of Sciences, Saint Petersburg, Russia and in Centro de Recursos de Biodiversidad Animal, University of Barcelona, Spain.

### Feather mite community construction and analyses

Here we examined the community structure of feather mite assemblages based on the presence-absence of feather mite species on each examined bird, and tested the influence of host phylogeny and geography on their composition and structure. For each host species, colonies belonging to the same archipelago were grouped to minimise differences in sample sizes among colonies (see columns “Colony” and “Area” in Supplementary Fig. S1 online). The number of European storm-petrels sampled was low and therefore, could not be included in these analyses.

We first described mite communities at different spatial scales. The terms for mite populations and communities follow Bush et al.^49^. Prevalence was calculated as the number of birds with a particular mite species divided by the number of examined birds. Overall mite prevalence was calculated for each host species and each colony/area using the statistical package Quantitative Parasitology 3.0^50^. Whittaker’s beta diversity, which describes the change in community composition at different scales, was calculated for each host species and each colony/area using PAST (version 2.17c)^51^. To evaluate the completeness of mite sampling, species accumulation curves were generated for each procellariiform species, pooling data from the different colonies/areas. Species accumulation curves represent the observed mite species number (S_obs_) and estimate the mite species number which would be collected as the number of samples approaches the population size. These analyses were conducted with PERMANOVA + for PRIMER v6^52^.

The influence of geography on the composition and structure of feather mite assemblages was tested at different spatial scales: large spatial scale (among the five regions mentioned above) and small spatial scale (among colonies within an area, e.g., Cape Verde Islands), while the influence of host taxonomy was tested at different host taxonomic levels: among the five host genera (*Calonectris*, *Puffinus*, *Bulweria, Pterodroma* and *Hydrobates*); among host species within a given genus (e.g., for *Calonectris* and *Puffinus* genera); and among individuals within a given host species (e.g., for Scopoli’s and the Cory’s shearwaters).

Similarity in mite community composition was examined at the infracommunity level. A mite infracommunity was defined as all symbionts of all species found on an individual host, whereas a component community includes all infracommunities within the same host population^49^. Mite infracommunities were used as replicate samples for community similarity analyses. For these analyses, we added a dummy species with value 1 to all host individuals to include those seabirds harbouring no feather mites.

We used *metaMDS* function of the vegan v2.5–4 R package to perform a non-metric multidimensional scaling (NMDS) to obtain an ordination of mite infracommunities for different host species and at different spatial scales (colony, area and region)^53^. This analysis was assuming Jaccard dissimilarities that were obtained with the *vegdist* function (binary = T). To visualize the ordinations, we used the phyloseq, ggplot2, and wesanderson R packages^54–56^. To assess the effects of host species and spatial scale on the composition and structure of mite communities, we conducted a permutational multivariate analysis of variance (PERMANOVA) with either host species, genus, or spatial scale as a fixed factor. For the factors host species and genus, we also performed an additional analysis in which we included the date of origin of each genus (estimated divergence time for that genus) to better account for the phylogenetic relatedness of the taxa. For example, when analyzing the effect of the host genus, we used the approximate date of origin of each genus as a fixed factor rather than genus identity. In addition, when analyzing the effect of the host species in the case of Cape Verde Islands, in PERMANOVA models, the date of origin of each genus was included as the first factor followed by host species identity. The date of origin of each genus (MYA) was obtained from TimeTree using the evolutionary timeline tool^57^. PERMANOVAs were then performed using the *adonis2* function from vegan on Jaccard distance matrices with 999 iterations. When a significant interaction between factors was detected, separate PERMANOVAs for each factor were performed, including post-hoc pairwise contrasts, (R package pairwiseAdonis v0.0.1), with a Bonferroni correction for multiple tests^58^. Finally, we used the vegan *meandist* function to obtain pairwise dissimilarities between samples.

To investigate the relationship between host phylogenetic relatedness and mite community similarity, we ran a mantel test, using the mantel function from vegan, and matrices of host phylogenetic distances (patristic) and mite dissimilarity (Jaccard index). The phylogenetic information for the hosts was obtained from BirdTree. Specifically, we downloaded 1,000 trees from the Ericson backbone tree and then summarized them by computing a single ultrametric 50% majority-rule consensus tree using SumTree v 4.1.0 in DendroPy v4.1.0, following Rubolini et al.^59^ (Supplementary Fig. S1 online). Three species (Cory´s shearwater, Macaronesian shearwater and Boyd’s shearwater) were included as polytomies (i.e., multiple branching from a single node) at the genus level as there was no phylogenetic information for them. We used the cophenetic.phylo function from ape to obtain the host's patristic distances matrix (Supplementary Table S2 online). Furthermore, given variation in sampling effort among bird species, we used rarefied mite dissimilarity matrices based on the bird species with the lowest number of mite samples (i.e., 31 samples) using the *rrarefy* function from vegan (Supplementary Table S3 online). Finally, in order to assess the significance of the correlation between pairwise variation in mite assemblages and geographical distance between sampling colonies/areas, we carried out a Mantel-type test (999 permutations) and calculated Spearman’s correlation coefficients using the RELATE routine in the PRIMER v6 package. All these analyses were based on host species that had been sampled in more than one colony/area with at least one infested host.

## Results

### Species identification and number of communities

On the 11 studied procellariiform species, we found 33 species of feather mites belonging to three families and eight genera: *Zachvatkinia, Rhinozachvatkinia*, *Promegninia* (Avenzoariidae), *Microspalax*, *Brephosceles*, *Plicatalloptes* (Alloptidae), *Ingrassia* and *Opetiopoda* (Xolalgidae) (Supplementary Tables S4, S5 online). Among the mite species found, 11 were undescribed species and six were recently described^43–45^.

Nine different mite species were collected from the Scopoli’s shearwaters and eight from the Cape Verde shearwaters and the Bulwer´s petrels (Table 2). For most host species, the species accumulation curves of mite species reached a plateau, except for the Cape Verde shearwaters and the Cape Verde petrels, indicating that mite species richness may be slightly underestimated for these hosts (see Supplementary Fig. S2 online).

At the host genus level, each avian genus generally harboured distinct feather mite species. However, three mite species (*M. brevipes*, *B. puffini* and *Plicatalloptes* sp.1) were found on both *Calonectris* and *Puffinus* shearwaters. Half of the feather mite genera (*Zachvatkinia*, *Microspalax*, *Brephosceles* and *Ingrassia*) co-occurred in multiple species of procellariiforms, three genera (*Rhinozachvatkinia, Promegninia* and *Plicatalloptes*) were restricted to two host genera, whereas *Opetiopoda* inhabited only one host species. Furthermore, each host genus/species carried only one mite species of a given genus, except *Microspalax* and *Brephosceles*. Thus, two different species of *Microspalax* inhabited Cory´s shearwaters, whereas two or more *Brephosceles* species co-occurred on the same host genus/species and even on the same individual host.

### Similarity patterns and factors shaping feather mite community structure

From the 964 seabirds sampled in this study, 684 were infested with at least one mite species. The host species with highest overall mite prevalence were Scopoli’s and Cory’s shearwaters (100%), Cape Verde shearwaters (92.2%) and Manx shearwaters (90.6%), whereas those with lowest prevalence were Band-rumped storm-petrels and Cape Verde petrels (44% and 36.4%, respectively) (Table 1). The global prevalence of each feather mite species inhabiting procellariform birds, calculated across the host species harbouring a given mite species, ranged between 1.3% for *M. pterodromae* and 69.5% for *Z. ovata* (Table 2).

**Table 1:** The number of feather mite species (prevalence—*P* %; mean and total species number; estimates for diversity) found on each seabird species and sampling site/area in the north-east Atlantic Ocean and Mediterranean Sea (N1 = number of examined birds, N2 = number of infested birds).

Bird species	Sampling site/area	N1	N2	Overall *P* % (N2/N1%) (95%CI)	Number of mite species/ individual (mean ± SD)	Total number of mite species	Whittaker (beta diversity)
*Bulweria bulwerii*	Azores Is	17	11	64.7 (40.6–83.4)	0.7 ± 0.6	2	0.8
	Canary Is	6	4	66.7 (27.1–93.7)	1.2 ± 1.0	6	2.4
	Cape Verde	81	53	65.4 (54.3–75.4)	1.2 ± 1.2	8	3.2
	Total	104	68	65.4 (55.8–74.1)	1.1 ± 1.2	8	3.6
Calonectris borealis*	Almeria	12	12	100.0 (75.5–100.0)	1.3 ± 0.5	3	1.3
	Azores Is	98	98	100.0 (96.1–100.0)	1.7 ± 1.0	9	4.2
	Berlengas	16	16	100.0 (79.1–100.0)	2.3 ± 1.2	7	2.0
	Canary Is	50	50	100.0 (92.5–100.0)	2.0 ± 1.0	7	2.5
	Madeira	34	34	100.0 (90.2–100.0)	2.1 ± 1.2	8	2.8
	Total	210	210	100.0 (98.2–100.0)	1.9 ± 1.1	9	3.8
Calonectris diomedea*	Balearic Is	37	37	100.0 (90.9–100.0)	1.8 ± 1.0	5	1.7
	Creta	5	5	100.0 (50.0–100.0)	2.2 ± 0.8	4	0.8
	Hyeres	4	4	100.0 (48.3–100.0)	1.8 ± 0.5	3	0.7
	Murcia	9	9	100.0 (67.7–100.0)	1.7 ± 0.7	4	1.4
	Zembra	15	15	100.0 (77.8–100.0)	1.9 ± 0.6	5	1.7
	Total	70	70	100.0 (94.6–100.0)	1.8 ± 0.9	5	1.7
*Calonectris edwardsii*	Cape Verde	64	59	92.2 (82.9–96.9)	2.8 ± 1.7	8	1.7
*Hydrobates castro*	Azores Is	9	9	100.0 (67.7–100.0)	1.0 ± 0.0	1	0.0
	Berlengas	2	2	100.0 (22.4–100.0)	1.0 ± 0.0	2	1.0
	Canary Is	1	1	100.0 (5.1–100.0)	1.0 ± 0.0	1	0.0
	Cape Verde	241	96	39.8 (33.8–46.3)	0.6 ± 0.9	5	2.1
	Madeira	6	6	100.0 (58.9–100.0)	1.2 ± 0.4	4	2.4
	Total	259	114	44.0 (38.0–50.2)	0.7 ± 0.9	5	2.3
*Puffinus baroli*	Azores Is	19	13	63.3 (39.2–82.4)	2.2 ± 1.4	6	1.8
	Canary Is	6	2	33.3 (6.3–72.9)	1.0 ± 1.7	4	0.3
	Total	25	15	56.0 (35.8–74.4)	2.3 ± 1.4	6	1.6
*Puffinus boydi*	Cape Verde	58	42	72.4 (59.5–83.0)	1.7 ± 1.6	6	1.6
*Puffinus puffinus*	Heimaey	11	10	90.9 (59.7–99.5)	2.6 ± 1.9	6	1.1
	Copeland	16	14	87.5 (62.8–97.7)	1.8 ± 1.2	5	1.4
	Halival-Rum	5	5	100.0 (50.0–100.0)	3.2 ± 1.1	6	0.9
	Total	32	29	90.6 (75.3–97.4)	2.3 ± 1.5	6	1.3
*Puffinus yelkouan*	Hyeres	29	25	86.2 (69.2–95.1)	1.5 ± 0.8	4	1.6
*Pterodroma feae*	Cape Verde	77	28	36.4 (26.0–48.0)	0.4 ± 0.6	3	1.7

The two *Calonectris* species sampled by visual inspection are marked with *. The 100% prevalence found in both *Calonectris* species is an artifact due to the sampling method in which only infested feather were collected (see methods).

**Table 2:** Feather mite species found on procellariform seabirds breeding in the north-east Atlantic Ocean and Mediterranean Sea and their global prevalence.

Mite species	Global prevalence	*C. diomedea* (70)	*C. borealis* (210)	*C. edwardsii* (64)	*P. puffinus* (32)	*P. yelkouan* (29)	*P. baroli* (25)	*P. boydi* (58)	*B. bulwerii* (104)	*P. feae* (77)	*H. castro* (259)
Fam. Avenzoariidae											
* Zachvatkinia ovata*	69.5	X	X	X							
* Zachvatkinia oceanodromae*	16.2										X
* Zachvatkinia* sp.1	45.8				X	X	X	X			
* Zachvatkinia* sp.2	33.7								X		
* Zachvatkinia* sp.3	13.0									X	
* Rhinozachvatkinia calonectris*	5.1		X	X							
* Promegninia calonectris*	2.9		X	X							
* Promegninia bulweriae*	2.9								X		
Fam. Alloptidae											
* Microspalax brevipes*	41.6	X	X	X	X		X	X			
* Microspalax ardennae*	17.1		X								
* Microspalax bulweriae*	9.6								X		
* Microspalax pterodromae*	1.3									X	
* Microspalax cymochoreae*	5.0										X
* Brephosceles puffini*	31.8	X	X	X	X	X	X	X			
* Brephosceles decapus*	17.0										X
* Brephosceles lanceolatus*	4.6										X
* Brephosceles disjunctus*	26.0									X	
* Brephosceles* sp.1	30.8								X		
* Brephosceles* sp.2	3.8								X		
* Brephosceles* sp.3	1.9								X		
* Brephosceles* sp.4	9.0	X	X	X							
* Brephosceles* sp.5	9.6				X		X	X			
* Plicatalloptes* sp.1	22.3	X	X	X	X	X	X	X			
Fam. Xolalgidae											
* Ingrassia calonectris*	14.2		X	X							
* Ingrassia dubinini*	30.5				X	X	X	X			
* Ingrassia micronota*	26.0								X		
* Ingrassia oceanodromae*	23.9										X
* Opetiopoda bulweriae*	5.8								X		

Band-rumped storm-petrel *Hydrobates pelagicus* was not included in this analysis. The sample size of each host species is indicated below its corresponding name.

**Figure 2:**
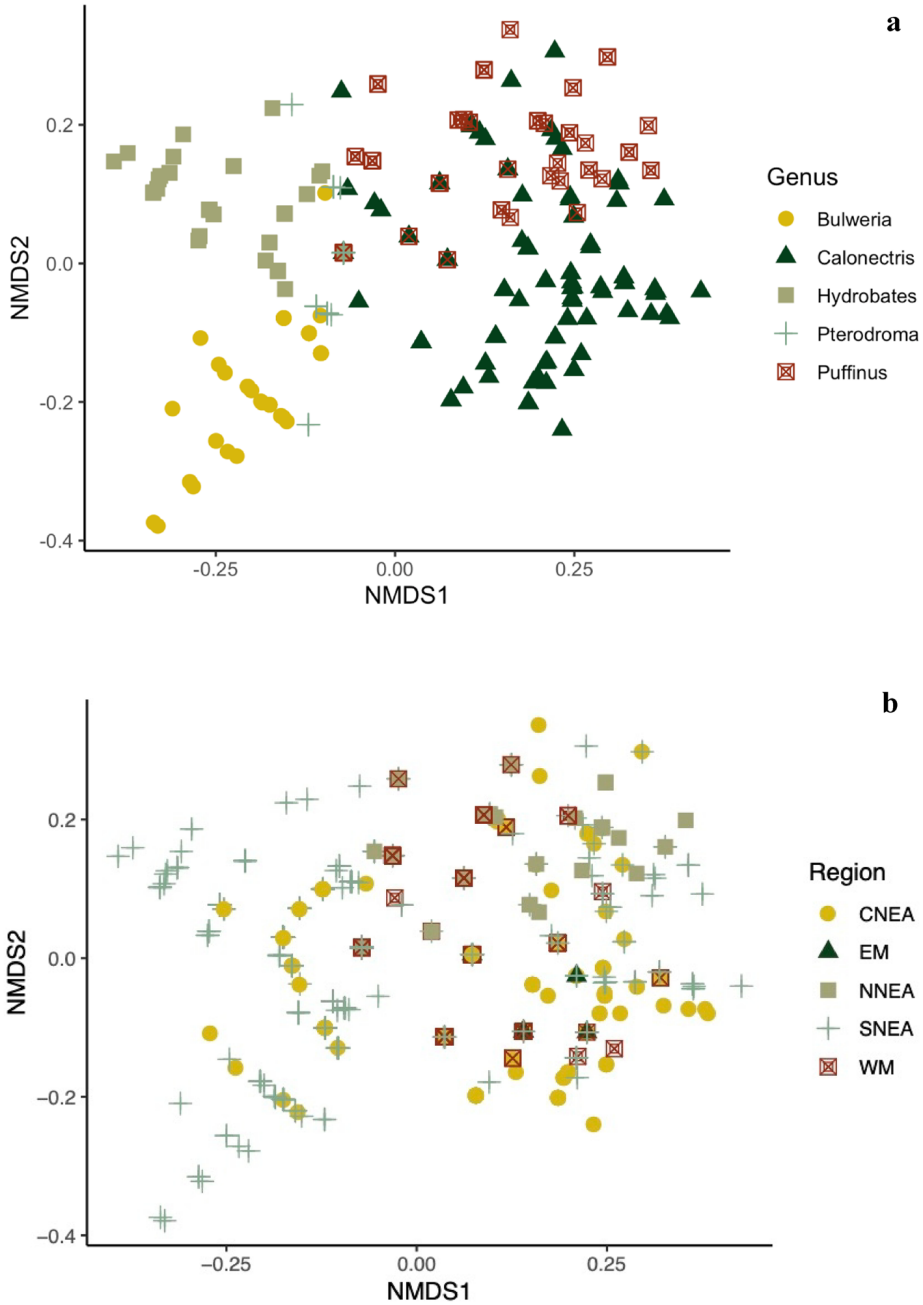
Non-metric multidimensional scaling ordination plot based on Jaccard similarities in mite species occurrence (data non-transformed, stress = 0.136) for (**a**) feather mite infracommunities of five procellariiform genera and (**b**) feather mite infracommunities of these five host genera presented as pooled data from the five sampled regions. Abbreviations of the geographical regions are: Western Mediterranean—WM, Eastern Mediterranean—EM, Northern NE Atlantic—NNEA, Central NE Atlantic—CNEA and Southern NE Atlantic—SNEA.

The non-metric multidimensional scaling (NMDS) ordination based on the relative similarities of feather mite infracommunities showed clear separation among the five seabird genera (*Calonectris*, *Puffinus*, *Bulweria, Pterodroma* and *Hydrobates*; Fig. 2a) with some overlap between *Calonectris* and *Puffinus* samples. In contrast, little separation was found among the five sampled regions (Fig. 2b). The PERMANOVA, used to assess the influence of host and region on the composition and structure of mite infracommunities, revealed a significant interaction between both factors (F = 9.15; *P* = 0.001). The separate PERMANOVAs for each factor indicated significant differences in feather mite community composition among both the five host genera (F = 103; *P* = 0.001) and the five geographic regions (F = 32.4; *P* = 0.001), but host genus explained more of the observed variation (30.1%) compared to geographic region (12.2%). An additional PERMANOVA analysis accounting for differences in the evolutionary age of each genus (i.e., using the date of origin of the genus as a factor instead of the genus identity) revealed similar results, with this factor explaining the 17.78% of the variance (F = 200, *P* = 0.001). Pairwise contrasts between all genera and regions indicated significant differences in mite community structure (host genera: F = 24.4–222, *P* = 0.01; regions: F = 2.02–80.4, *P* < 0.05), except for comparisons between the regions Western Mediterranean—Eastern Mediterranean and Central NE Atlantic—Eastern Mediterranean (*P* > 0.05; Fig. 2b).

**Figure 3:**
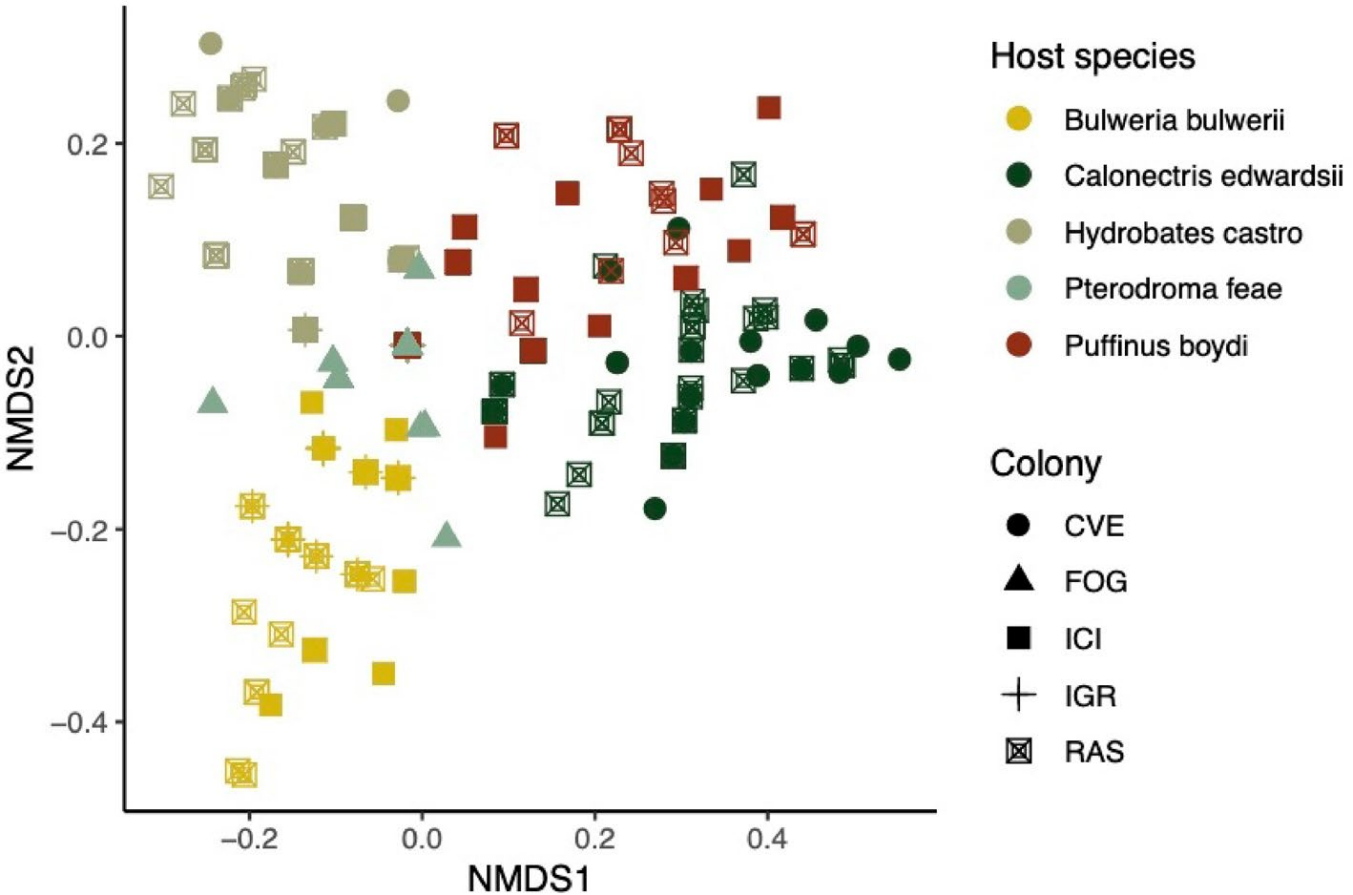
Non-metric multidimensional scaling ordination plot based on Jaccard similarities (data non-transformed, stress = 0.103) of mite infracommunities for five procellariiform species breeding in different colonies of the Cape Verde Islands. Each host species and sampled colony is represented by its own colours and icons, respectively. Abbreviations of the colonies are: Curral Velho—CVE, Fogo—FOG, Ilheu Cima—ICI, Ilheu Grande—IGR, Raso—RAS.

At a smaller geographic scale, the non-metric multidimensional scaling (NMDS) ordination on mite infracommunities of the five host species breeding in the Cape Verde Islands showed a clear separation by host species, regardless of colony location (Fig. 3). Similarly, we found that closely related hosts had similar mite communities (rho = 0.802, *P* = 0.002). The PERMANOVA revealed an interaction between host species and sampling colony, (F = 4.17; *P* = 0. 001); a re-analysis with each separate factor indicated significant differences in mite community composition among the five host species (F = 50.7; *P* = 0.001) and among the five colonies (Raso, Curral Velho, Fogo, Ilheu Cima and Ilheu Grande) (F = 11.3; *P* = 0.001). Host species again explained a higher percentage of the observed variation compared to geography (28.14% compared to 8%). The same result was found after accounting for differences in the evolutionary age of each genus (16.3% variance; F = 39.1; *P* = 0.001). All pairwise contrasts for both factors were significant (host species: F = 23.6–107, *P* = 0.01; colony: F = 3.63–20.8, *P* < 0.05). The highest similarity in mite community composition was found between Cape Verde petrels and Band-rumped storm-petrels (63.4%) and the lowest between Cape Verde shearwaters and Bulwer’s petrels (25.4%).

**Figure 4:**
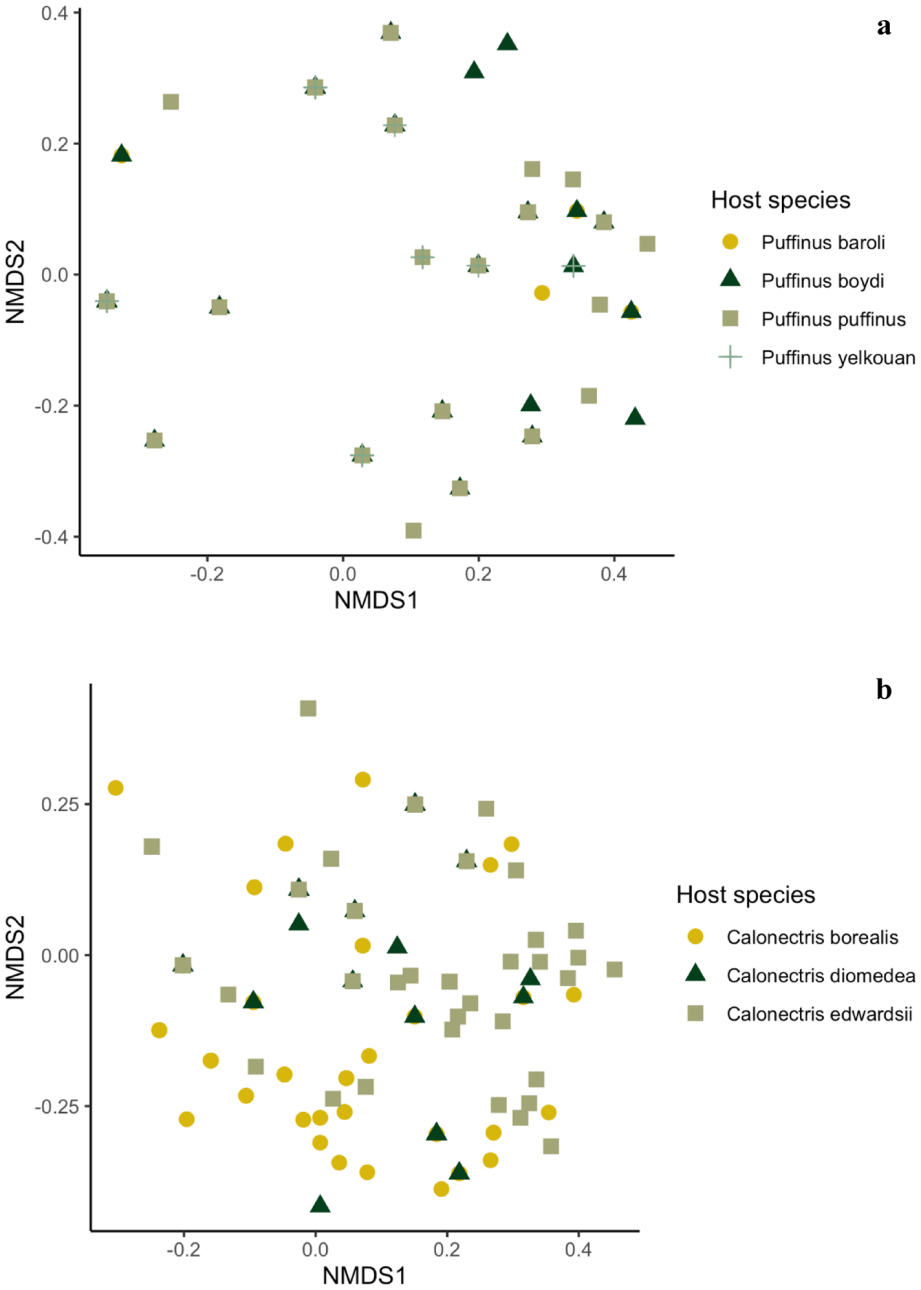
Non-metric multidimensional scaling ordination plot based on Jaccard similarities in mite species occurrence (data non-transformed, stress = 0.138) for (**a**) mite infracommunities of four species of *Puffinus* and (**b**) three species of *Calonectris*. Although the graph suggests no clear structure of mite infracommunities by host species, the PERMANOVA indicated significant differences in feather mite infracommunity composition in both genera. Data were pooled when host species were sampled in different areas (see Table 1).

Regarding differences in feather mite infracommunities among host species within *Puffinus* and *Calonectris* genera, visual inspection of the NMDS ordination did not suggest clear structure of mite infracommunities by host species (Fig. 4). However, the PERMANOVA indicated significant differences in feather mite infracommunity composition among species within each genus (*Puffinus* species F = 3.17; *P* = 0.001; Fig. 4a and *Calonectris* species F = 15.1; *P* = 0.001; Fig. 4b). Pairwise comparisons revealed significant differences for both *Calonectris* (all comparisons: F = 7.27–24.8, *P* < 0.003) and *Puffinus* species (only for Mediterranean and Boyd's and Manx shearwaters: F = 1.44–5.22, *P* < 0.05). Average similarities among individuals of the same *Puffinus* species ranged from 44.3% for the Manx shearwaters to 57.8% for the Mediterranean shearwaters, whereas the highest similarity between two host species was 50.7% (Macaronesian and Mediterranean shearwaters). Calculated similarities among individuals within the three *Calonectris* species ranged from 44.8% (Cape Verde shearwaters) to 54.9% (Scopoli’s shearwaters), whereas the highest similarity between two host species was 50.8% (Cory’s and Scopoli´s shearwaters).

**Figure 5:**
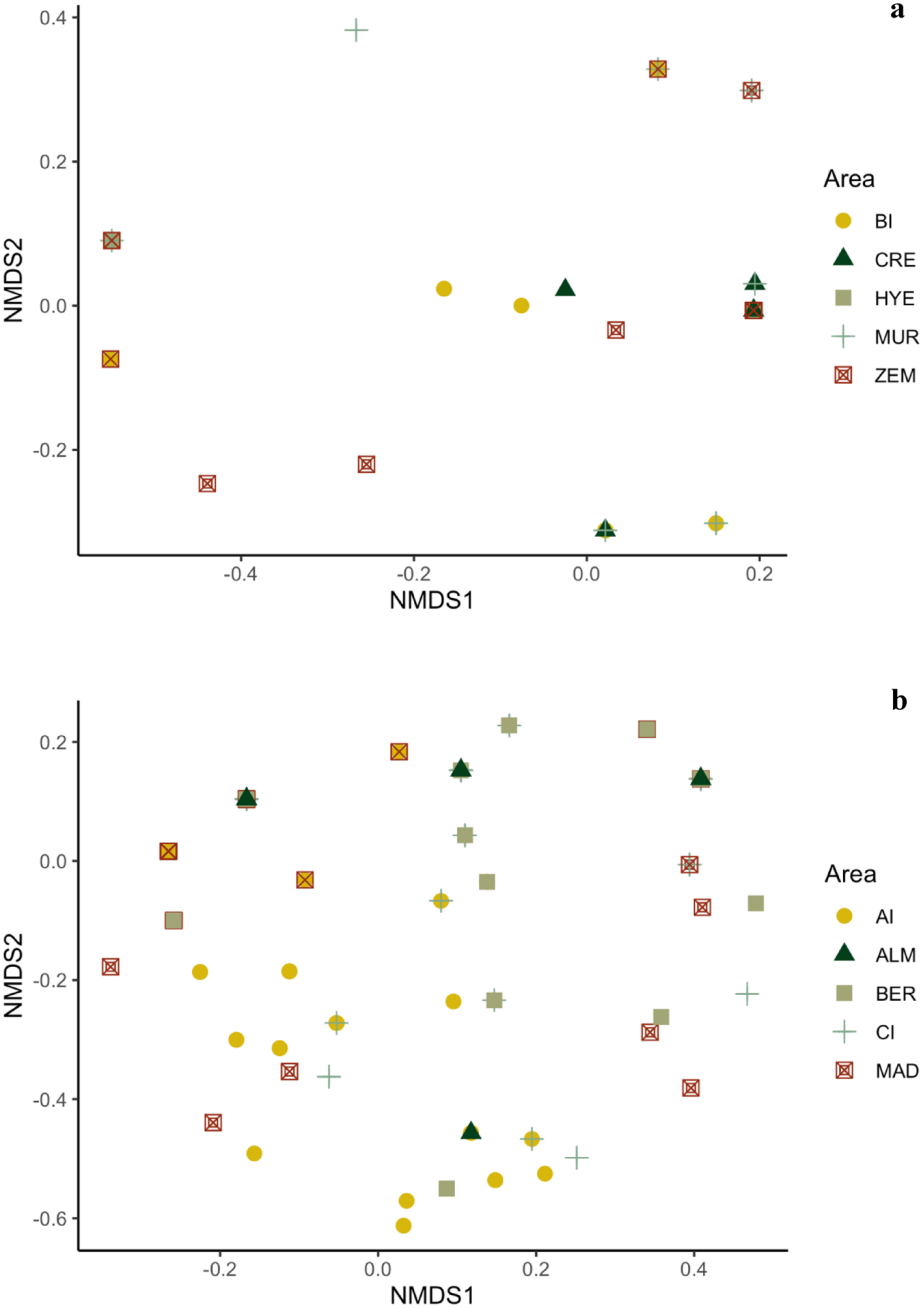
Non-metric multidimensional scaling ordination plot based on Jaccard similarities (data non-transformed, stress = 0.100) for mite infracommunities of (**a**) Scopoli´s shearwaters and (**b**) Cory’s shearwaters across their corresponding sampling colonies/areas. No clear separation of mite infracommunities between colonies was observed for both host species. Abbreviations of the geographical sampling sites/areas are: Balearic Islands—BI, Crete—CRE, Hyeres—HYE, Murcia—MUR, Zembra—ZEM.

Detailed analyses of the influence of geography on the feather mite communities within and among host populations in the two wide-spread *Calonectris* species, the Scopoli´s and the Cory´s shearwaters, showed noticeable variability in the structure of mite assemblages. The non-metric multidimensional scaling (NMDS) indicated a lack of structuring of mite infracommunities by colony/area for the Scopoli’s shearwaters (Fig. 5a), a result further supported by the PERMANOVA (F = 1.28; *P* = 0.232). The pairwise contrast between colonies/areas also revealed no differences for this host species. Similarities in infracommunities within colonies/areas were always higher than 50.0%, while similarities between colonies/areas ranged from 44.2% (Murcia–Zembra) to 70.2% (Creta–Hyeres). In the case of Cory’s shearwaters, the NMDS ordination showed some geographic separation of mite communities (Fig. 5b), and the PERMANOVA indicated significant geographic structuring (F = 12.8; *P* = 0.001), with colony explaining 20% of the variation. Mite community structure differed significantly between the five sampled areas (F = 1.55–30.1, *P* < 0.05), except for comparisons between Canary Islands, Almeria and Berlengas. Similarities in infracommunities within colonies/areas ranged between 45.1% (Berlengas) and 63.0% (Azores Islands), while average similarities between colonies/areas ranged from 39.6% (Madeira – Berlengas) to 59.3% (Almeria–Canary Islands). Geographic distance among colonies/areas correlated positively with the similarity of the mite communities on Cory’s shearwaters, although the correlation coefficient was relatively low (RELATE: *rho* = 0.176, *P* = 0.0001).

## Discussion

In this study we used a large survey of seabird feather mite assemblages to examine the relative roles of host phylogeny and geography in shaping the communities of these symbionts. We found that similarity in mite assemblages was determined to a great extent by host phylogenetic relatedness, with weak or negligible community structure among host species from the same host genera or among host individuals from the same host species sampled across distant localities.

The 11 procellariiform species breeding in the north-east Atlantic Ocean and the Mediterranean Sea harboured at least 33 species belonging to eight mite genera, half of which were either undescribed or recently described species. Species belonging to the genera *Brephosceles*, *Zachvatkinia*, *Microspalax* and *Ingrassia* were the most frequently reported from these seabird hosts. At least three feather mite species were found per seabird species, with the highest mite species richness reported for *Calonectris* shearwaters and Bulwer´s petrels. Species accumulation curves of mite richness reached the asymptote in most host species, indicating that we detected all common mite species harboured by these birds. However, this was not the case for two seabird species (Cape Verde shearwater and Cape Verde petrel), suggesting that further sampling may be needed to reveal the entire feather mite richness in these hosts. These findings highlight the still incomplete knowledge we currently have of seabird acarofauna.

To identify factors driving community structure of feather mites on procellariiform birds, we examined the presence/absence of mite species at different host taxonomic and geographic scales. Based on community similarity analyses, we found high specificity at the host genus level at both small (within the Cape Verde Islands) and large (among the five defined regions) geographic scales. Within the Cape Verde Islands, feather mite communities were clearly structured by host genus. That is, even when seabird species of different genera breed sympatrically in close physical contact (i.e., sharing the same breeding habitat or even burrow), mite assemblages differed. This result is in line with the genetic analyses performed on these feather mite communities^42^. However, at this spatial scale, the different seabird genera were represented by single species, such that host specificity of feather mites at host species level cannot be ruled out. At a larger geographic scale, three out of five studied seabird genera (*Calonectris*, *Puffinus* and *Hydrobates*) were represented by several host species sampled across several archipelagos. In this case, different host species from the same genus showed similar mite assemblages, and each genus harboured essentially distinct feather mite species, confirming that feather mite communities are essentially structured at the host genus level. Despite this general pattern, two sister genera of shearwaters, *Calonectris* and *Puffinus*, with nine and six mite species respectively, shared three mite species (*Brephosceles puffini*, *Microspalax brevipes* and *Plicatalloptes* sp.1), indicating that some mite species can inhabit closely-related host genera^23^. These results suggest that the high degree of specificity at the host species level advocated in some previous studies^19,23^ may be inflated by incomplete sampling of closely related species. Indeed, some of the mite species detected in the present study were first records for the examined host species, but were previously found in other closely related host species. One should therefore be careful about concluding on the degree of feather mite specificity before all closely related host species have been thoroughly sampled.

At a lower taxonomic level, that is at the host species level (i.e., among host species within *Calonectris* and *Puffinus* genera), we also found small but significant dissimilarities in feather mite communities. The Cory´s, Cape Verde and Scopoli’s shearwaters hosted nine, eight and five mite species, respectively, of which, a set of five mite species was shared among all *Calonectris* species and a distinct set of three mite species was shared only by Cory’s and Cape Verde shearwaters. The three *Calonectris* species have parapatric distributions. Direct contact between individuals of Cory’s and Scopoli’s shearwaters is largely limited to the Chafarinas Islands, a breeding locality situated on the boundary of their geographic ranges (nominally the Strait of Gibraltar), with some occasional reports of shearwaters visiting and even breeding on other localities within the breeding range of the other species^60^. Neither of these species shares a breeding colony with the Cape Verde shearwaters, although some individuals of the latter species are occasionally found visiting breeding colonies of Cory’s shearwaters in the Canary Islands^61^. Similarly, in the closely related *Puffinus* species, two species, Manx and Boyd’s shearwaters, share the same set of six mite species, whereas the other two, Macaronesian and Yelkouan shearwaters, host only a subset of three species. These results show that even when contact zones still exist between recently diverged host species, geography can limit mite transmission and lead to some, although weak, structuring of their assemblages. In contrast, among the ten mite species found to inhabit the two *Hydrobates* species, the Band-rumped and the European storm-petrels, only one mite species (*B. lanceolatus*) was shared. The two *Hydrobates* species are more phylogenetically distant than the three *Calonectris* species or the four *Puffinus* species^62,63^, suggesting a decrease in the similarity of mite communities with host phylogenetic distance^5,7,9^.

After controlling for host effects, some geographic differences in feather mite communities were detected among the five defined regions. However, this geographic pattern may largely reflect the parapatric distribution of host species from the same genus rather than geography per se. Indeed, no geographic structure of feather mite communities was observed for Scopoli’s shearwaters in the Mediterranean Sea, and only a weak signal was found among localities for the widely distributed Cory’s shearwaters. These findings contrast results from other systems^6,8,16,17^, where parasite community similarity decreased with geographic distance. However, these studies mainly involved endoparasites from freshwater and terrestrial environments, and the decay in similarity was primarily linked to differences in host traits (e.g., vagility, body weight or trophic level) and/or environmental gradients. Our host-symbiont system clearly differs from other biological models by two main factors: feather mite life histories and extreme host vagility. These factors could explain the weak influence of geographic distances on seabird feather mite community structure. Feather mites spend their entire life cycle on the bird’s body and may therefore, be highly tolerant to rapid changes in environmental conditions across the geographic range of the host species^19,20^. In addition, the extreme vagility of seabirds can promote feather mite dispersal among host populations even at large spatial scales, thus weakening differentiation of feather mite assemblages with distance. Our results are in accordance with a recent study, which also reported strong host-associated structuring and no geographic signature of feather mite assemblages across avian taxa, even at a continental scale^23^.

In this study, we examined differences in mite community assemblages based on presence/absence data and did not consider the relative abundance of the different mite species. It is possible that community structure may differ in this respect among geographic locations or at lower host taxonomic levels. Studying feather mite communities in this way is nonetheless difficult without a strong standardization of the methods used for collecting mites. Indeed, even with the dust-ruffling method, differences in weather conditions during field sampling can modify the efficiency of the collection method (i.e., wind). In conclusion, among the highly diverse feather mite community harboured by procellariiform seabirds we found that feather mite communities showed a high degree of specificity at the level of the host genus, at both small and large geographical scales; each seabird genus harbouring essentially distinct feather mite communities, with some exceptions in phylogenetically related seabird genera *Calonectris* and *Puffinus*. These results suggest that specificity at the host species level advocated in previous studies could have resulted from incomplete sampling of closely related host species. Thus, a thorough sampling of feather mites from all closely related host species is required when concluding on the degree of host specificity of these ectosymbionts. Geographic structure of feather mite communities was weak at best, likely due to seabird vagility that provides dispersal opportunities for feather mites. Geographic distance showed little association with mite community similarity, and its effect was negligible compared to other factors, suggesting that symbionts confined to the host body are highly tolerant to environmental gradients. Overall, our results highlight the key importance of host phylogeny in explaining major patterns of community structure in ectosymbionts, but supports host genus-specific mite assemblages rather than species-specific. However, molecular studies of mite communities may reveal potential barriers to feather mite dispersal that are not observed by species presence/absence data alone.

## Supplementary Information

Supplementary Information.

## Data Availability

The dataset generated and analyzed during the current study is available from the corresponding author on reasonable request. Slide mounted mites are available at Zoological Institute of the Russian Academy of Sciences, Saint Petersburg, Russia and in Centro de Recursos de Biodiversidad Animal, University of Barcelona, Spain.
