# Supplementary material for: Diversity and structure of feather mite communities on seabirds from the north–east Atlantic and Mediterranean Sea
Source: Sci Rep. 2023 Mar 23;13:4793. doi: 10.1038/s41598-023-30858-8 (PMC10036324; doi:10.1038/s41598-023-30858-8)

**Supplementary Information**

**Table S1.** Sample size of host examined and harbouring feather mites by seabird species and locality.

| **Country** | **Colony** | **Code** | **Area** | **Code** | **Region** | **Code** | **Lat/Long** | **Host scientific name** | **Number of examined birds** | **Number of infested birds** |  |
| --- | --- | --- | --- | --- | --- | --- | --- | --- | --- | --- | --- |
| Spain | Murcia | MUR |  |  | Western Mediterranean | WM | 37^o^34'59''N/00^o^58'59''W | *Calonectris diomedea* | 9 | 9 | |
| Spain | Ibiza | IBZ | Balearic Is. | BI | Western Mediterranean | WM | 38^o^57'42''N/01^o^11'53''E | *Calonectris diomedea* | 21 | 21 | |
|  |  |  |  |  |  |  |  | *Hydrobates pelagicus* | 30 | 20 | |
| Spain | Mallorca | MAL | Balearic Is. | BI | Western Mediterranean | WM | 39^o^39'58''N/02^o^34'53''E | *Calonectris diomedea* | 9 | 9 | |
| Spain | Menorca | MEN | Balearic Is. | BI | Western Mediterranean | WM | 39^o^48'07''N/04^o^17'16''E | *Calonectris diomedea* | 7 | 7 | |
|  |  |  |  |  |  |  |  | *Hydrobates pelagicus* | 3 | 1 | |
| France | Hyeres | HYE |  |  | Western Mediterranean | WM | 43^o^00'32''N/06^o^12'38''E | *Calonectris diomedea* | 4 | 4 | |
|  |  |  |  |  |  |  |  | *Puffinus yelkouan* | 29 | 25 | |
| Tunisia | Zembra | ZEM |  |  | Western Mediterranean | WM | 37^o^07'33''N/10^o^48'10''E | *Calonectris diomedea* | 15 | 15 | |
| Spain | Almeria | ALM |  |  | Western Mediterranean | WM | 37^o^20'56''N/01^o^39'02''W | *Calonectris borealis* | 12 | 12 | |
|  |  |  |  |  |  |  |  | *Hydrobates pelagicus* | 1 | 1 | |
| Greece | Crete | CRE |  |  | Eastern Mediterranean | EM | 35^o^36'38''N/23^o^34'49''E | *Calonectris diomedea* | 5 | 5 | |
| Iceland | Heimaey | HEI |  |  | Northern NE Atlantic | NNEA | 63^o^27'00''N/20^o^15'00''W | *Puffinus puffinus* | 11 | 10 | |
| Ireland | Copeland | COP |  |  | Northern NE Atlantic | NNEA | 54^o^40'29''N/05^o^31'44''W | *Puffinus puffinus* | 16 | 14 | |
|  |  |  |  |  |  |  |  | *Hydrobates pelagicus* | 2 | 2 | |
| Scotland | Halival-Rum | RUM |  |  | Northern NE Atlantic | NNEA | 57^o^00'05''N/06^o^19'21''W | *Puffinus puffinus* | 5 | 5 | |
| Portugal | Madeira | MAD |  |  | Central NE Atlantic | CNEA | 32^o^20'40''N/16^o^29'08''W | *Calonectris borealis* | 34 | 34 | |
|  |  |  |  |  |  |  |  | *Hydrobates castro* | 6 | 6 | |
| Portugal | Berlengas | BER |  |  | Central NE Atlantic | CNEA | 39^o^24'32''N/09^o^29'38''W | *Calonectris borealis* | 16 | 16 | |
|  |  |  |  |  |  |  |  | *Hydrobates castro* | 2 | 2 | |
| Spain | Gran Canaria | GCA | Canary Is. | CI | Central NE Atlantic | CNEA | 27^o^50'40''N/15^o^47'19''W | *Calonectris borealis* | 30 | 30 | |
| Spain | Lanzarote | LAN | Canary Is. | CI | Central NE Atlantic | CNEA | 29^o^17'29''N/13^o^31'57''W | *Calonectris borealis* | 12 | 12 | |
|  |  |  |  |  |  |  |  | *Puffinus baroli* | 6 | 2 | |
|  |  |  |  |  |  |  |  | *Bulweria bulwerii* | 3 | 2 | |
|  |  |  |  |  |  |  |  | *Hydrobates castro* | 1 | 1 | |
| Spain | Tenerife | TEN | Canary Is. | CI | Central NE Atlantic | CNEA | 28^o^26'59''N/16^o^13'59''W | *Calonectris borealis* | 8 | 8 | |
| Spain | La Palma | PAL | Canary Is. | CI | Central NE Atlantic | CNEA | 28^o^48'54''N/17^o^45'54''W | *Bulweria bulwerii* | 3 | 2 | |
| Portugal | Corvo | COR | Azores Is. | AI | Central NE Atlantic | CNEA | 39^o^40'28''N/31^o^06'21''W | *Calonectris borealis* | 15 | 15 | |
| Portugal | Flores | FLO | Azores Is. | AI | Central NE Atlantic | CNEA | 39^o^22'29''N/31^o^11'50''W | *Calonectris borealis* | 14 | 14 | |
| Portugal | Faial | FAI | Azores Is. | AI | Central NE Atlantic | CNEA | 38^o^31'27''N/28^o^44'48''W | *Calonectris borealis* | 12 | 12 | |
| Portugal | Graciosa | GRA | Azores Is. | AI | Central NE Atlantic | CNEA | 39^o^03'20''N/27^o^57'17''W | *Calonectris borealis* | 23 | 23 | |
|  |  |  |  |  |  |  |  | *Hydrobates castro* | 4 | 4 | |
| Portugal | Sao Miguel | SMI | Azores Is. | AI | Central NE Atlantic | CNEA | 37^o^43'02''N/25^o^25'59''W | *Calonectris borealis* | 5 | 5 | |
| Portugal | Santa Maria | SMA | Azores Is. | AI | Central NE Atlantic | CNEA | 36^o^56'31''N/25^o^10'17''W | *Calonectris borealis* | 29 | 29 | |
|  |  |  |  |  |  |  |  | *Puffinus baroli* | 19 | 13 | |
|  |  |  |  |  |  |  |  | *Bulweria bulwerii* | 17 | 11 | |
|  |  |  |  |  |  |  |  | *Hydrobates castro* | 5 | 5 | |
|  |  |  |  |  |  |  |  |  |  |  | |
| Cape Verde | Raso | RAS | Cape Verde | CV | Southern NE Atlantic | SNEA | 16^o^36'36''N/24^o^36'00''W | *Calonectris edwardsii* | 44 | 39 | |
|  |  |  |  |  |  |  |  | *Puffinus boydi* | 29 | 23 | |
|  |  |  |  |  |  |  |  | *Bulweria bulwerii* | 31 | 23 | |
|  |  |  |  |  |  |  |  | *Hydrobates castro* | 139 | 74 | |
| Cape Verde | Curral Velho | CVE | Cape Verde | CV | Southern NEAtlantic | SNEA | 15^o^58'10''N/22^o^47'22''W | *Calonectris edwardsii* | 20 | 20 | |
|  |  |  |  |  |  |  |  | *Hydrobates castro* | 36 | 14 | |
| Cape Verde | Fogo | FOG | Cape Verde | CV | Southern NE Atlantic | SNEA | 14^o^59'28''N/24^o^22'12''W | *Pterodroma feae* | 77 | 28 | |
| Cape Verde | Ilhéu Cima | ICI | Cape Verde | CV | Southern NE Atlantic | SNEA | 14^o^58'11''N/24^o^38'21''W | *Puffinus boydi* | 29 | 19 | |
|  |  |  |  |  |  |  |  | *Bulweria bulwerii* | 30 | 15 | |
|  |  |  |  |  |  |  |  | *Hydrobates castro* | 54 | 7 | |
| Cape Verde | Ilhéu Grande | IGR | Cape Verde | CV | Southern NE Atlantic | SNEA | 14^o^58'09''N/24^o^41'20''W | *Bulweria bulwerii* | 20 | 15 | |
|  |  |  |  |  |  |  |  | *Hydrobates castro* | 12 | 1 | |

**Fig. S1.** Host tree used for the phylogenetic comparative methods (see Methods for more details). Values indicate ML bootstrap support.

**
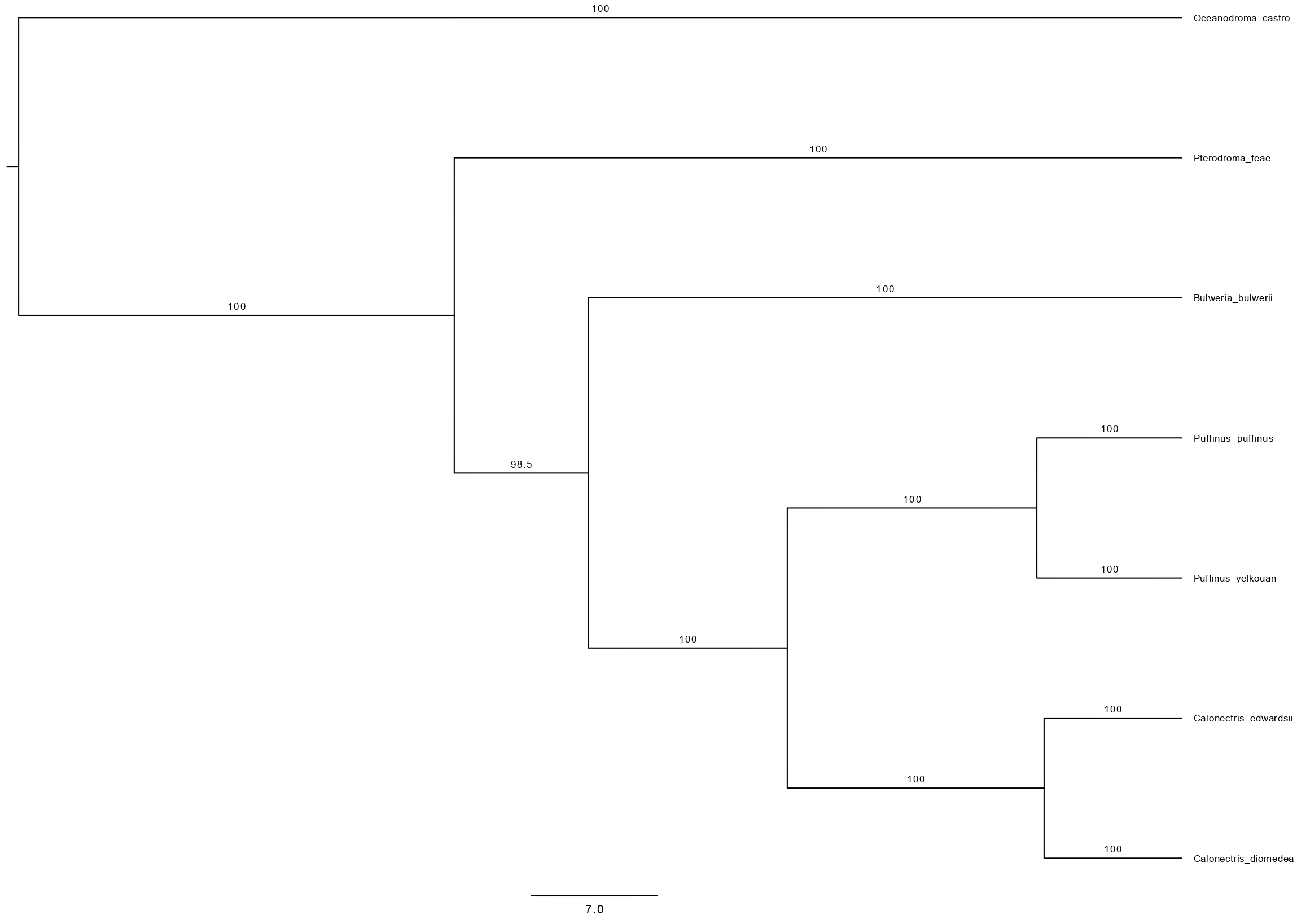
**

**Table S2.** Pairwise host patristic distances matrix.

| "Hydrobates_castro","Pterodroma_feae","Bulweria_bulwerii","Puffinus_boydi","Puffinus_baroli","Puffinus_puffinus","Puffinus_yelkouan","Calonectris borealis","Calonectris_edwardsii","Calonectris_diomedea" | | | | | | | | | | | | | | | |
| --- | --- | --- | --- | --- | --- | --- | --- | --- | --- | --- | --- | --- | --- | --- | --- |
| Hydrobates_castro,0,129.18,129.18,129.18,129.18,129.18,129.18,129.18,129.18,129.18 | | | | | | | | | | | | | | |  |
| Pterodroma_feae,129.18,0,80.8,80.8,80.8,80.8,80.8,80.8,80.8,80.8 | | | | | | | | | | | | | |  |  |
| Bulweria_bulwerii,129.18,80.8,0,65.9,65.9,65.9,65.9,65.9,65.9,65.9 | | | | | | | | | | | | | |  |  |
| Puffinus_boydi,129.18,80.8,65.9,0,16.09,16.09,16.09,43.81,43.81,43.81 | | | | | | | | | | | | | |  |  |
| Puffinus_baroli,129.18,80.8,65.9,16.09,0,16.09,16.09,43.81,43.81,43.81 | | | | | | | | | | | | | |  |  |
| Puffinus_puffinus,129.18,80.8,65.9,16.09,16.09,0,16.09,43.81,43.81,43.81 | | | | | | | | | | | | | | |  |
| Puffinus_yelkouan,129.18,80.8,65.9,16.09,16.09,16.09,0,43.81,43.81,43.81 | | | | | | | | | | | | | | |  |
| Calonectris borealis,129.18,80.8,65.9,43.81,43.81,43.81,43.81,0,15.29,15.29 | | | | | | | | | | | | | |  |  |
| Calonectris_edwardsii,129.18,80.8,65.9,43.81,43.81,43.81,43.81,15.29,0,15.29 | | | | | | | | | | | | | | |  |
| Calonectris_diomedea,129.18,80.8,65.9,43.81,43.81,43.81,43.81,15.29,15.29,0 | | | | | | | | | | | | | | |  |
|  |  |  |  |  |  |  |  |  |  |  |  |  |  |  |  |

**Table S3.** Pairwise feather mite community dissimilarity matrix.

| "Hydrobates_castro","Pterodroma_feae","Bulweria_bulwerii","Puffinus_boydi","Puffinus_baroli","Puffinus_puffinus","Puffinus_yelkouan","Calonectris borealis","Calonectris_edwardsii","Calonectris_diomedea" | | |  |  |  |  |  |
| --- | --- | --- | --- | --- | --- | --- | --- |
| Hydrobates_castro,0,1,1,1,1,1,1,1,1,1 |  |  |  |  |  |  |  |
| Pterodroma_feae,1,0,1,1,1,1,1,1,1,1 |  |  |  |  |  |  |  |
| Bulweria_bulwerii,1,1,0,1,1,1,1,1,1,1 |  |  |  |  |  |  |  |
| Puffinus_boydi,1,1,1,0,0,0,0.333,0.7,0.7,0.625 | | | |  |  |  |  |
| Puffinus_baroli,1,1,1,0,0,0,0.333,0.7,0.7,0.625 | | | |  |  |  |  |
| Puffinus_puffinus,1,1,1,0,0,0,0.333,0.7,0.7,0.625 | | | |  |  |  |  |
| Puffinus_yelkouan,1,1,1,0.333,0.333,0.333,0,0.778,0.778,0.714 | | | | | | | |
| Calonectris_borealis,1,1,1,0.7,0.7,0.7,0.778,0,0.444,0.5 | | | | |  |  |  |
| Calonectris_edwardsii,1,1,1,0.7,0.7,0.7,0.778,0.444,0,0.286 | | | | | | |  |
| Calonectris_diomedea,1,1,1,0.625,0.625,0.625,0.714,0.5,0.286,0 | | | | | |  |  |

**Table S4.** Taxonomic diversity of feather mites harboured by 11 procellariiform species breeding in the Mediterranean Sea and northeastern Atlantic Ocean ordered by host species. Species nominated as sp.1, sp.2, etc. are undescribed new species. * represents new host records.

| **Host species** | **Feather mite species** | **Colony** | **Region** |
| --- | --- | --- | --- |
| *Calonectris diomedea* | *Zachvatkinia ovata* | Murcia, Ibiza, Mallorca, Menorca, Hyeres, Crete, Zembra | Mediterranean |
|  | *Microspalax brevipes* | Murcia, Ibiza, Mallorca, Menorca, Hyeres, Crete, Zembra | Mediterranean |
|  | *Brephosceles puffini*^*^ | Murcia, Ibiza, Menorca, Hyeres, Crete, Zembra | Mediterranean |
|  | *Brephosceles* sp.4 | Ibiza, Zembra | Mediterranean |
|  | *Plicatalloptes* sp.1 | Murcia, Ibiza, Mallorca, Menorca, Crete, Zembra | Mediterranean |
| *Calonectris borealis* | *Zachvatkinia ovata* | Almeria | Mediterranean |
|  |  | Madeira, Berlengas, Gran Canaria, Lanzarote, Tenerife, Corvo, Flores, Faial, Graciosa, Sao Miguel, Santa Maria | NE Atlantic |
|  | *Microspalax brevipes* | Almeria | Mediterranean |
|  |  | Madeira, Berlengas, Gran Canaria, Lanzarote, Tenerife, Corvo, Flores, Faial, Graciosa, Sao Miguel, Santa Maria | NE Atlantic |
|  | *Microspalax ardennae* | Madeira, Berlengas, Corvo, Flores, Graciosa, Santa Maria | NE Atlantic |
|  | *Brephosceles puffini*^*^ | Almeria | Mediterranean |
|  |  | Madeira, Berlengas, Gran Canaria, Lanzarote, Tenerife, Corvo, Flores, Faial, Graciosa, Sao Miguel, Santa Maria | NE Atlantic |
|  | *Brephosceles* sp.4 | Madeira, Berlengas, Gran Canaria, Lanzarote, Corvo, Flores, Faial, Graciosa, Sao Miguel | NE Atlantic |
|  | *Plicatalloptes* sp.1 | Madeira, Berlengas, Gran Canaria, Lanzarote, Tenerife, Corvo, Flores, Faial, Graciosa, Santa Maria | NE Atlantic |
|  | *Ingrassia calonectris* | Madeira, Lanzarote, Flores, Santa Maria | NE Atlantic |
|  | *Rhinozachvatkinia calonectris* | Corvo, Flores, Graciosa, Santa Maria | NE Atlantic |
|  | *Promegninia calonectris* | Madeira, Berlengas, Gran Canaria, Corvo | NE Atlantic |
| *Calonectris edwardsii* | *Zachvatkinia ovata* | Raso, Curral Velho | NE Atlantic |
|  | *Microspalax brevipes* | Raso, Curral Velho | NE Atlantic |
|  | *Brephosceles puffini* | Raso, Curral Velho | NE Atlantic |
|  | *Brephosceles* sp.4 | Raso, Curral Velho | NE Atlantic |
|  | *Plicatalloptes* sp.1 | Raso, Curral Velho | NE Atlantic |
|  | *Ingrassia calonectris* | Raso, Curral Velho | NE Atlantic |
|  | *Rhinozachvatkinia calonectris* | Raso, Curral Velho | NE Atlantic |
|  | *Promegninia calonectris* | Raso | NE Atlantic |
| *Puffinus puffinus* | *Zachvatkinia* sp.1 | Heimaey, Copeland, Halival-Rum | NE Atlantic |
|  | *Microspalax brevipes*^*^ | Heimaey, Copeland, Halival-Rum | NE Atlantic |
|  | *Brephosceles puffini*^*^ | Heimaey, Copeland, Halival-Rum | NE Atlantic |
|  | *Brephosceles* sp.5 | Heimaey, Halival-Rum | NE Atlantic |
|  | *Plicatalloptes* sp.1 | Heimaey, Copeland, Halival-Rum | NE Atlantic |
|  | *Ingrassia dubinini*^*^ | Heimaey, Copeland, Halival-Rum | NE Atlantic |
| *Puffinus boydi* | *Zachvatkinia* sp.1 | Raso, Ilhéu Cima | NE Atlantic |
|  | *Microspalax brevipes* | Raso, Ilhéu Cima | NE Atlantic |
|  | *Brephosceles puffini* | Raso, Ilhéu Cima | NE Atlantic |
|  | *Brephosceles* sp.5 | Raso | NE Atlantic |
|  | *Plicatalloptes* sp.1 | Raso, Ilhéu Cima | NE Atlantic |
|  | *Ingrassia dubinini* | Raso, Ilhéu Cima | NE Atlantic |
| *Puffinus baroli* | *Zachvatkinia* sp.1 | Lanzarote | NE Atlantic |
|  | *Microspalax brevipes*^*^ | Lanzarote | NE Atlantic |
|  | *Brephosceles puffini*^*^ | Lanzarote | NE Atlantic |
|  | *Ingrassia dubinini*^*^ | Lanzarote | NE Atlantic |
| *Puffinus yelkouan* | *Zachvatkinia* sp.1 | Hyeres | Mediterranean |
|  | *Brephosceles puffini*^*^ | Hyeres | Mediterranean |
|  | *Ingrassia dubinini*^*^ | Hyeres | Mediterranean |
| *Pterodroma feae* | *Zachvatkinia* sp.3 | Fogo | NE Atlantic |
|  | *Microspalax pterodromae* | Fogo | NE Atlantic |
|  | *Brephosceles disjunctus* | Fogo | NE Atlantic |
| *Bulweria bulwerii* | *Zachvatkinia* sp.2 | La Palma, Santa Maria, Raso, Ilhéu Cima, Ilhéu Grande | NE Atlantic |
|  | *Microspalax bulweriae* | La Palma, Raso, Ilhéu Cima, Ilhéu Grande | NE Atlantic |
|  | *Brephosceles* sp.1 | Lanzarote, Santa Maria, Raso, Ilhéu Cima, Ilhéu Grande | NE Atlantic |
|  | *Brephosceles* sp.2 | Raso, Ilhéu Cima | NE Atlantic |
|  | *Brephosceles* sp.3 | Ilhéu Cima | NE Atlantic |
|  | *Ingrassia micronota* | Lanzarote, Raso, Ilhéu Cima, Ilhéu Grande | NE Atlantic |
|  | *Opetiopoda bulweriae* | Lanzarote, Raso | NE Atlantic |
|  | *Promegninia bulweriae* | La Palma, Raso, Ilhéu Cima | NE Atlantic |
| *Hydrobates castro* | *Zachvatkinia oceanodromae* | Madeira, Berlengas, Lanzarote, Graciosa, Santa Maria, Raso, Curral Velho, Ilhéu Cima, Ilhéu Grande | NE Atlantic |
|  | *Microspalax cymochoreae* | Madeira, Raso, Curral Velho, Ilhéu Cima | NE Atlantic |
|  | *Brephosceles decapus* | Madeira, Berlengas, Raso, Curral Velho | NE Atlantic |
|  | *Brephosceles lanceolatus* | Raso, Curral Velho, Ilhéu Cima | NE Atlantic |
|  | *Ingrassia oceanodromae* | Madeira, Raso, Curral Velho, Ilhéu Cima | NE Atlantic |
| *Hydrobates pelagicus* | *Zachvatkinia hidrobatidii* | Ibiza | Mediterranean |
|  | *Rhinozachvatkinia* sp.1 | Ibiza | Mediterranean |
|  | *Brephosceles pelagicus* | Ibiza, Menorca | Mediterranean |
|  | *Brephosceles lanceolatus*^*^ | Ibiza, Menorca | Mediterranean |
|  | *Brephosceles* sp.6 | Ibiza | Mediterranean |
|  | *Ingrassia oceanica* | Ibiza | Mediterranean |

**Table S5**. Taxonomic diversity of feather mites harboured by 11 procellariiform species breeding in the Mediterranean Sea and northeastern Atlantic Ocean ordered by feather mite species. Species nominated as sp.1, sp.2, etc. are undescribed new species. * represents new host records.

| **Feather mite species** | **Host species** | **Colony** | **Region** |
| --- | --- | --- | --- |
| **Fam. Avenzoariidae** |  |  |  |
| *Zachvatkinia ovata* | *Calonectris diomedea* | Murcia (Spain) | Mediterranean |
|  |  | Ibiza, Mallorca, Menorca (Balearic Is. - Spain) | Mediterranean |
|  |  | Hyeres (France) | Mediterranean |
|  |  | Crete (Greece) | Mediterranean |
|  |  | Zembra (Tunisia) | Mediterranean |
|  | *Calonectris borealis* | Almeria (Spain) | Mediterranean |
|  |  | Madeira (Portugal) | NE Atlantic |
|  |  | Berlengas (Portugal) | NE Atlantic |
|  |  | Gran Canaria, Lanzarote, Tenerife (Canary Is. - Spain) | NE Atlantic |
|  |  | Corvo, Flores, Faial, Graciosa, Sao Miguel, Santa Maria (Azores Is. - Spain) | NE Atlantic |
|  | *Calonectris edwardsii* | Raso, Curral Velho (Cape Verde) | NE Atlantic |
| *Zachvatkinia oceanodromae* | *Hydrobates castro* | Madeira (Portugal) | NE Atlantic |
|  |  | Berlengas (Portugal) | NE Atlantic |
|  |  | Lanzarote (Canary Is. - Spain) | NE Atlantic |
|  |  | Graciosa, Santa Maria (Azores Is. - Spain) | NE Atlantic |
|  |  | Raso, Curral Velho, Ilhéu Cima, Ilhéu Grande (Cape Verde) | NE Atlantic |
| *Zachvatkinia hidrobatidii* | *Hydrobates pelagicus* | Ibiza (Balearic Is. - Spain) | Mediterranean |
| *Zachvatkinia* sp.1 | *Puffinuspuffinus* | Copeland (Northern Ireland)  Heimaey (Iceland)  Halival-Rum (Scotland) | NE Atlantic  NE Atlantic  NE Atlantic |
|  | *Puffinus boydi* | Raso, Ilhéu Cima (Cape Verde) | NE Atlantic |
|  | *Puffinus baroli* | Lanzarote (Canary Is. - Spain) | NE Atlantic |
|  | *Puffinus yelkouan* | Hyeres (France) | Mediterranean |
| *Zachvatkinia* sp.2 | *Bulweria bulwerii* | La Palma (Canary Is. - Spain)  Santa Maria (Azores Is. - Spain)  Raso, Ilhéu Cima, Ilhéu Grande (Cape Verde) | NE Atlantic  NE Atlantic  NE Atlantic |
| *Zachvatkinia* sp.3 | *Pterodroma feae* | Fogo (Cape Verde) | NE Atlantic |
| *Rhinozachvatkinia calonectris* | *Calonectris borealis* | Corvo, Flores, Graciosa, Santa Maria (Azores Is. - Spain) | NE Atlantic |
|  | *Calonectris edwardsii* | Raso, Curral Velho (Cape Verde) | NE Atlantic |
| *Rhinozachvatkinia* sp.1 | *Hydrobates pelagicus* | Ibiza (Balearic Is. - Spain) | Mediterranean |
| *Promegninia calonectris* | *Calonectris borealis* | Madeira (Portugal) | NE Atlantic |
|  |  | Berlengas (Portugal) | NE Atlantic |
|  |  | Gran Canaria (Canary Is. - Spain) | NE Atlantic |
|  |  | Corvo (Azores Is. - Spain) | NE Atlantic |
|  | *Calonectris edwardsii* | Raso (Cape Verde) | NE Atlantic |
| *Promegninia bulweriae* | *Bulweria bulwerii* | La Palma (Canary Is. - Spain)  Raso, Ilhéu Cima (Cape Verde) | NE Atlantic  NE Atlantic |
| **Fam. Alloptidae** |  |  |  |
| *Microspalax brevipes* | *Calonectris diomedea* | Murcia (Spain) | Mediterranean |
|  |  | Ibiza, Mallorca, Menorca (Balearic Is. - Spain) | Mediterranean |
|  |  | Hyeres (France) | Mediterranean |
|  |  | Crete (Greece) | Mediterranean |
|  |  | Zembra (Tunisia) | Mediterranean |
|  | *Calonectris borealis* | Almeria (Spain) | Mediterranean |
|  |  | Madeira (Portugal) | NE Atlantic |
|  |  | Berlengas (Portugal) | NE Atlantic |
|  |  | Gran Canaria, Lanzarote, Tenerife (Canary Is. - Spain) | NE Atlantic |
|  |  | Corvo, Flores, Faial, Graciosa,  Sao Miguel, Santa Maria (Azores Is. - Spain) | NE Atlantic |
|  | *Calonectris edwardsii* | Raso, Curral Velho (Cape Verde) | NE Atlantic |
|  | *Puffinus puffinus*^*^ | Copeland (Northern Ireland)  Heimaey (Iceland)  Halival-Rum (Scotland) | NE Atlantic  NE Atlantic  NE Atlantic |
|  | *Puffinus boydi* | Raso, Ilhéu Cima (Cape Verde) | NE Atlantic |
|  | *Puffinus baroli*^*^ | Lanzarote (Canary Is. - Spain) | NE Atlantic |
| *Microspalax ardennae* | *Calonectris borealis* | Madeira (Portugal) | NE Atlantic |
|  |  | Berlengas (Portugal) | NE Atlantic |
|  |  | Corvo, Flores, Graciosa, Santa Maria (Azores Is. - Spain) | NE Atlantic |
| *Microspalax bulweriae* | *Bulweria bulwerii* | La Palma (Canary Is. - Spain) | NE Atlantic |
|  |  | Raso, Ilhéu Cima, Ilhéu Grande (Cape Verde) | NE Atlantic |
| *Microspalax pterodromae* | *Pterodroma feae* | Fogo (Cape Verde) | NE Atlantic |
| *Microspalax cymochoreae* | *Hydrobates castro* | Madeira (Portugal)  Raso, Curral Velho, Ilhéu Cima (Cape Verde) | NE Atlantic  NE Atlantic |
| *Brephosceles puffini* | *Calonectris diomedea*^*^ | Murcia (Spain) | Mediterranean |
|  |  | Ibiza, Menorca (Balearic Is. - Spain) | Mediterranean |
|  |  | Hyeres (France) | Mediterranean |
|  |  | Crete (Greece) | Mediterranean |
|  |  | Zembra (Tunisia) | Mediterranean |
|  | *Calonectris borealis*^*^ | Almeria (Spain) | Mediterranean |
|  |  | Madeira (Portugal) | NE Atlantic |
|  |  | Berlengas (Portugal) | NE Atlantic |
|  |  | Gran Canaria, Lanzarote, Tenerife (Canary Is. - Spain) | NE Atlantic |
|  |  | Corvo, Flores, Faial, Graciosa, Sao Miguel, Santa Maria (Azores Is. - Spain) | NE Atlantic |
|  | *Calonectris edwardsii* | Raso, Curral Velho (Cape Verde) | NE Atlantic |
|  | *Puffinus puffinus*^*^ | Copeland (Northern Ireland)  Heimaey (Iceland)  Halival-Rum (Scotland) | NE Atlantic  NE Atlantic  NE Atlantic |
|  | *Puffinusboydi* | Raso, Ilhéu Cima (Cape Verde) | NE Atlantic |
|  | *Puffinus baroli*^*^ | Lanzarote (Canary Is. - Spain) | NE Atlantic |
|  | *Puffinus yelkouan*^*^ | Hyeres (France) | Mediterranean |
| *Brephosceles decapus* | *Hydrobates castro* | Madeira (Portugal) | NE Atlantic |
|  |  | Berlengas (Portugal) | NE Atlantic |
|  |  | Raso, Curral Velho (Cape Verde) | NE Atlantic |
| *Brephosceles pelagicus* | *Hydrobates pelagicus* | Ibiza, Menorca (Balearic Is. - Spain) | Mediterranean |
| *Brephosceles lanceolatus* | *Hydrobates castro* | Raso, Curral Velho, Ilhéu Cima (Cape Verde) | NE Atlantic |
|  | *Hydrobates pelagicus*^*^ | Ibiza, Menorca (Balearic Is. - Spain) | Mediterranean |
| *Brephosceles disjunctus* | *Pterodroma feae*^*^ | Fogo (Cape Verde) | NE Atlantic |
| *Brephosceles* sp.1 | *Bulweria bulwerii* | Lanzarote (Canary Is. - Spain) | NE Atlantic |
|  |  | Santa Maria (Azores Is. - Spain) | NE Atlantic |
|  |  | Raso, Ilhéu Cima, Ilhéu Grande (Cape Verde) | NE Atlantic |
| *Brephosceles* sp.2 | *Bulweria bulwerii* | Raso, Ilhéu Cima (Cape Verde) | NE Atlantic |
| *Brephosceles* sp.3 | *Bulweria bulwerii* | IlhéuCima (Cape Verde) | NE Atlantic |
| *Brephosceles* sp.4 | *Calonectris diomedea* | Ibiza (Balearic Is. - Spain) | Mediterranean |
|  |  | Zembra (Tunisia) | Mediterranean |
|  | *Calonectris borealis* | Madeira (Portugal) | NE Atlantic |
|  |  | Berlengas (Portugal) | NE Atlantic |
|  |  | Gran Canaria, Lanzarote (Canary Is. - Spain) | NE Atlantic |
|  |  | Corvo, Flores, Faial, Graciosa, Sao Miguel (Azores Is. - Spain) | NE Atlantic |
|  | *Calonectris edwardsii* | Raso, Curral Velho (Cape Verde) | NE Atlantic |
| *Brephosceles* sp.5 | *Puffinus puffinus* | Heimaey (Iceland)  Halival-Rum (Scotland) | NE Atlantic  NE Atlantic |
|  | *Puffinus boydi* | Raso (Cape Verde) | NE Atlantic |
| *Brephosceles* sp.6 | *Hydrobates pelagicus* | Ibiza (Balearic Is. - Spain) | Mediterranean |
| *Plicatalloptes* sp.1 | *Calonectris diomedea* | Murcia (Spain) | Mediterranean |
|  |  | Ibiza, Mallorca, Menorca (Balearic Is. - Spain) | Mediterranean |
|  |  | Crete (Greece) | Mediterranean |
|  |  | Zembra (Tunisia) | Mediterranean |
|  | *Calonectris borealis* | Madeira (Portugal) | NE Atlantic |
|  |  | Berlengas (Portugal) | NE Atlantic |
|  |  | Gran Canaria, Lanzarote, Tenerife (Canary Is. - Spain) | NE Atlantic |
|  |  | Corvo, Flores, Faial, Graciosa, Santa Maria (Azores Is. - Spain) | NE Atlantic |
|  | *Calonectris edwardsii* | Raso, Curral Velho (Cape Verde) | NE Atlantic |
|  | *Puffinus puffinus* | Copeland (Northern Ireland) | NE Atlantic |
|  |  | Heimaey (Iceland) | NE Atlantic |
|  |  | Halival-Rum (Scotland) | NE Atlantic |
|  | *Puffinus boydi* | Raso, Ilhéu Cima (Cape Verde) | NE Atlantic |
| **Fam. Xolalgidae** |  |  |  |
| *Opetiopoda bulweriae* | *Bulweria bulwerii* | Lanzarote (Canary Is. - Spain) | NE Atlantic |
|  |  | Raso (Cape Verde) | NE Atlantic |
| *Ingrassia calonectris* | *Calonectris borealis* | Madeira (Portugal) | NE Atlantic |
|  |  | Lanzarote (Canary Is. - Spain) | NE Atlantic |
|  |  | Flores, Santa Maria (Azores Is. - Spain) | NE Atlantic |
|  | *Calonectris edwardsii* | Raso, Curral Velho (Cape Verde) | NE Atlantic |
| *Ingrassia dubinini* | *Puffinus puffinus*^*^ | Heimaey (Iceland) | NE Atlantic |
|  |  | Copeland (Northern Ireland) | NE Atlantic |
|  |  | Halival-Rum (Scotland) | NE Atlantic |
|  | *Puffinus boydi* | Raso, Ilhéu Cima (Cape Verde) | NE Atlantic |
|  | *Puffinus baroli*^*^ | Lanzarote (Canary Is. - Spain) | NE Atlantic |
|  | *Puffinus yelkouan*^*^ | Hyeres (France) | Mediterranean |
| *Ingrassia micronota* | *Bulweria bulwerii* | Lanzarote (Canary Is. - Spain) | NE Atlantic |
|  |  | Raso, Ilhéu Cima, Ilhéu Grande (Cape Verde) | NE Atlantic |
| *Ingrassia oceanodromae* | *Hydrobates castro* | Madeira (Portugal) | NE Atlantic |
|  |  | Raso, Curral Velho, Ilhéu Cima (Cape Verde) | NE Atlantic |
| *Ingrassia oceanica* | *Hydrobates pelagicus* | Ibiza (Balearic Is. - Spain) | Mediterranean |

**Figure S2.** Mite species accumulation curves for ten procellariiform host species. Data is pooled over all sampled areas. Species observed (Sobs) - black triangles. X axis represents the total number of samples examined for a given seabird species and Y axis represents the number of mite species found on a given host species. BULBUL – *Bulweria bulwerii,* CALBOR – *Calonectris borealis*, CALDIO – *Calonectris diomedea*, CALEDW – *Calonectris edwardsii*, HYDCAS – *Hydrobates castro,* PUFBAR – *Puffinus baroli*, PUFBOY – *Puffinus boydi*, PUFPUF – *Puffinus puffinus,* PUFYEL – *Puffinus yelkouan*, PTEFEA – *Pterodroma feae.*


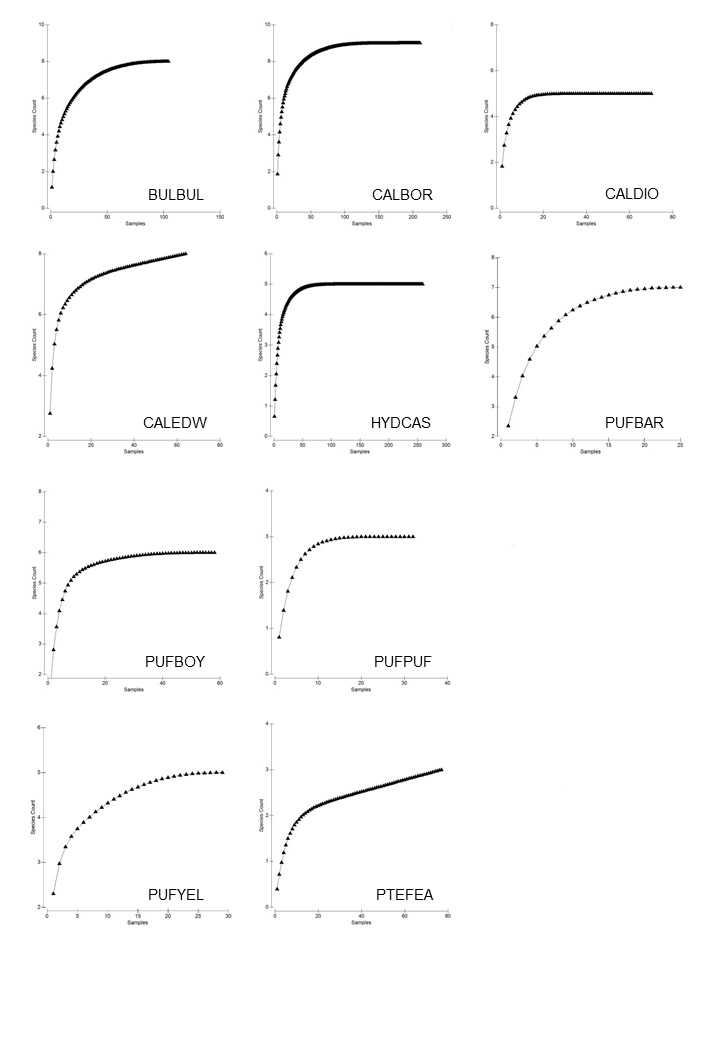

Supplement: Supplementary file 1 — Supplementary Information. [file 41598_2023_30858_MOESM1_ESM.docx]
